# The effect of estrogenic compounds on psychosis-like behaviour in female rats

**DOI:** 10.1371/journal.pone.0193853

**Published:** 2018-03-26

**Authors:** Alyssa Sbisa, Maarten van den Buuse, Andrea Gogos

**Affiliations:** 1 Hormones in Psychiatry Laboratory, Florey Institute of Neuroscience and Mental Health, Parkville, VIC, Australia; 2 School of Psychology and Public Health, La Trobe University, Bundoora, VIC, Australia; 3 Department of Pharmacology, University of Melbourne, Parkville, VIC, Australia; 4 The College of Public Health, Medical and Veterinary Sciences, James Cook University, Townsville, QLD, Australia; Chiba Daigaku, JAPAN

## Abstract

17β-estradiol treatment has shown benefit against schizophrenia symptoms, however long-term use may be associated with negative side-effects. Selective estrogen receptor modulators, such as raloxifene and tamoxifen, have been proposed as suitable alternatives to 17β-estradiol. An isomer of 17β-estradiol, 17α-estradiol, is considered less carcinogenic, and non-feminising in males, however little is known about its potential as a treatment for schizophrenia. Moreover, the mechanism underlying the therapeutic action of estrogens remains unclear. We aimed to investigate the ability of these estrogenic compounds to attenuate psychosis-like behaviour in rats. We used two acute pharmacologically-induced assays of psychosis-like behaviour: psychotomimetic drug-induced hyperlocomotion and disruption of prepulse inhibition (PPI). Female Long Evans rats were either intact, ovariectomised (OVX), or OVX and chronically treated with 17β-estradiol, 17α-estradiol, raloxifene or tamoxifen. Only 17β-estradiol treatment attenuated locomotor hyperactivity induced by the indirect dopamine receptor agonist, methamphetamine. 17β-estradiol- and tamoxifen-treated rats showed attenuated methamphetamine- and apomorphine (dopamine D1/D2 receptor agonist)-induced disruption of PPI. Raloxifene-treated rats showed attenuated apomorphine-induced PPI disruption only. Baseline PPI was significantly reduced following OVX, and this deficit was reversed by all estrogenic compounds. Further, PPI in OVX rats was increased following administration of apomorphine. This study confirms a protective effect of 17β-estradiol in two established animal models of psychosis, while tamoxifen showed beneficial effects against PPI disruption. In contrast, 17α-estradiol and raloxifene showed little effect on dopamine receptor-mediated psychosis-like behaviours. This study highlights the utility of some estrogenic compounds to attenuate psychosis-like behaviour in rats, supporting the notion that estrogens have therapeutic potential for psychotic disorders.

## Introduction

A large body of literature demonstrates the utility of the ‘female’ sex steroid, estrogen, more specifically 17β-estradiol (17β), as novel treatment for schizophrenia [1–3]. Preclinical and clinical studies have demonstrated the beneficial effects of treatment with estrogens for schizophrenia, particularly against the positive symptoms [1,2,4]. However, due to the risk of peripheral side effects [5,6], including some cancers and feminising effects in males, the investigation of alternative estrogenic compounds is warranted.

The selective estrogen receptor modulator (SERM), raloxifene (RAL), is typically used in the treatment of osteoporosis [7]. RAL has exhibited beneficial effects across the spectrum of schizophrenia symptoms in the clinical population [8–10]. For example, in postmenopausal women with schizophrenia, RAL administered in conjunction with antipsychotic treatment, improved negative symptoms [9] as well as positive symptoms of the illness [11]. RAL has also demonstrated favourable effects on verbal memory and attention in men and women with schizophrenia [10,12]. Another SERM, tamoxifen (TAM), is used as an anti-estrogen therapy for breast cancer [7], however it has also demonstrated efficacy in preclinical models of schizophrenia-like symptoms [13], and in women with acute bipolar affective disorder [14]. 17α-estradiol (17α), an isomer of 17β, is another estrogenic compound recently highlighted as a neuroactive steroid [15–17] and may be a potential therapeutic candidate in schizophrenia. Compared to 17β, 17α is considered to weakly bind to estrogen receptor (ER)-α and ER-β, and preferentially binds to a membrane estrogen receptor (ER-X) [18,19]. 17α has no uterotrophic effects, reducing the likelihood of estrogen-induced endometrial cancer [17,20]. Previous research has primarily investigated the effect of 17α *in vitro* [21] and in animal models of learning and memory [22], depression [23], and anxiety [15]; however, its effects on psychosis-like behaviour is unknown. Further, the mechanism underlying the therapeutic action of SERMs and 17α remains unclear.

Two of the most widely used assays of psychosis-like behaviour in rodents are disruption of prepulse inhibition of the acoustic startle response (PPI) and psychotomimetic drug-induced locomotor hyperactivity [24,25]. PPI is a cross-species measure of sensorimotor gating and deficits in PPI are present in patients with schizophrenia including untreated patients, and those treated with typical antipsychotics [26,27]. Experimental animals exhibit PPI deficits following treatment with dopamine receptor agonists [28]. Psychotomimetic drug-induced locomotor hyperactivity is a behavioural test used to model the brain mechanisms involved in psychosis, particularly psychotic agitation/excitement [24,25].

Previously, we found that ovariectomised (OVX) rats treated chronically with 17β, RAL or TAM showed attenuated PPI disruptions induced by administration of the dopamine D1/D2 receptor agonist, apomorphine [13]. In the current study, we extended this work by examining the effect of various estrogenic compounds on PPI disruption and locomotor hyperactivity induced by the monoamine releaser and indirect dopamine receptor agonist, methamphetamine. Thus, we assessed the effect of chronic treatment with the estradiols, 17β and 17α, and the SERMs, RAL and TAM, on PPI disruption induced by apomorphine and methamphetamine, and on methamphetamine-induced locomotor hyperactivity.

## Materials and methods

### Animals

Sixty-four Long Evans (LE) rats (Florey Institute of Neuroscience and Mental Health, VIC, Australia) were housed at La Trobe University (VIC, Australia) in groups of four in individually-ventilated cages (Tecniplast, Italy), with *ad libitum* access to standard pellet food and tap water. The rats were maintained on a 12h light–dark cycle (lights on at 0700h), at an ambient temperature of 22 ± 2°C. All surgical techniques, treatments and experimental protocols were approved by the La Trobe University Animal Ethics Committee and conducted in accordance with the Australian Code of Practice for the Care and Use of Animals for Scientific Purposes (1990) set out by the National Health and Medical Research Council of Australia.

### Surgery

Ovariectomy surgery was performed as described previously [29]. Briefly, 12 week old rats were anaesthetised using an isoflurane/oxygen gas mixture and received a subcutaneous (s.c.) injection of 5 mg/kg of the non-steroidal, anti-inflammatory analgesic, carprofen (Rimadyl®; Heriot AgVet, VIC, Australia). A small dorsal midline incision was made through the skin, followed by an incision through the abdominal wall, and the ovaries were bilaterally located and removed. Intact rats were sham-operated (SHAM); they received all procedures except the ovaries were not excised.

Rats were randomly allocated to 6 groups (*n* = 10–11 per group; Table 1): OVX with a s.c. implant (Dow Corning, I.D. 1.98 mm, O.D. 3.18 mm; Futuremedics, VIC, Australia) filled with 100% crystalline 17β (5 mm length, ~30 mg per implant; Cayman Chemical Company, MI, USA), 17α (5 mm length, ~25 mg per implant; Sigma Chemical Company, MO, USA), RAL (2 x 20 mm length, ~45 mg per implant; Toronto Research Chemicals, ON, Canada) or TAM (2 x 20 mm length, ~65 mg per implant; Toronto Research Chemicals). Untreated OVX rats and SHAM rats received an empty implant. These implant sizes were based on literature [30,31] and our previous findings [13], and were aimed at producing pharmacologically active doses [13,29]. Implants remained in the rat for approximately 6 weeks. At the end of the experiment, rats were euthanized and uterus and pituitary weights were recorded to confirm effective hormone treatment [13,32]. One 17β-treated animal was excluded from data analysis due to extremely low uterus weight indicating ineffective hormone treatment.

**Table 1:** Body weight (BW), uterus weight (UW), and pituitary gland weight (PW) of female rats.

Group	n	Surgery BW	Weight gain	UW	UW/BW	PW
SHAM	11	183 ± 3	15 ± 3	284 ± 34**	1.43 ± 0.16**	12 ± 0.6
OVX	11	182 ± 4	23 ± 5	112 ± 9	0.55 ± 0.05	10 ± 0.3
17β	10	173 ± 5	20 ± 4	521 ± 34**	2.66 ± 0.16**	42 ± 4.0*
17α	10	179 ± 5	23 ± 5	131 ± 0	0.66 ± 0.04	10 ± 0.6
RAL	10	180 ± 5	23 ± 6	121 ± 1	0.60 ± 0.04	9 ± 0.7
TAM	11	182 ± 3	0 ± 2**	127 ± 5	0.71 ± 0.05	8 ± 0.6

Body weight (BW, g), uterus weight (UW, mg) and pituitary gland weight (PW, mg) are expressed as mean ± SEM. Weight gain is the difference between body weight on the day of surgery and body weight at the end of experimentation. Rats were intact (SHAM), ovariectomised (OVX), or OVX rats treated with 17β-estradiol (17β), 17α-estradiol (17α), raloxifene (RAL) or tamoxifen (TAM).

** *p* ≤ 0.001

* *p* ≤ 0.05, compared to OVX group.

### Behavioural experiments

Locomotor activity was measured using eight automated photocell chambers (ENV-520, MED Associates, VT, USA), as previously described [32]. Briefly, the position of the rat within the chamber was detected via 16 evenly spaced infrared sources and sensors on each of the four sides of the monitor, which measured x, y, and z axes movements. During the experiment, rats were placed in the locomotor chamber for 30 min to allow habituation; the rats were subsequently injected and locomotor activity was recorded for a further 90 min.

PPI of the acoustic startle response was measured with eight automated startle chambers (SR-Lab; San Diego Instruments, San Diego, CA, USA) as previously described [29]. Briefly, rats were placed individually into a transparent Plexiglas cylinder in a sound-attenuating cabinet. The PPI session comprised 80 trials presented with variable intervals (8–27 s), including 32 startle pulse-alone trials (4 blocks of eight 115 dB trials) and 40 prepulse–pulse trials. Prepulse–pulse trials consisted of a prepulse of an intensity 2, 4, 8, 12 or 16 dB above the 70 dB background (eight per intensity), followed 100 ms later by the startle pulse. Startle data were measured using all 4 blocks of pulse-alone trials. The %PPI was calculated as [(pulse-alone trials startle amplitude minus prepulse–pulse trials startle amplitude) / (pulse-alone trials startle amplitude)] × 100%. The middle 16 pulse-alone trials were used to calculate %PPI. Three rats were deemed outliers and excluded from PPI analysis (1 OVX, 1 RAL, 1 17α). These 3 rats had extremely low average baseline PPI; specifically average PPI <13%, which was greater than 2 times the standard deviation of that group.

At least ten days after surgery, rats were tested for PPI after administration of saline, 1 mg/kg of methamphetamine, and 0.1 mg/kg of apomorphine. Following a one-week washout period, the same rats were tested for locomotor hyperactivity following administration of saline or 1 mg/kg methamphetamine. In a pseudo-randomised, crossover protocol, rats received all drug treatments with at least a 3-day washout period between each testing session. This allowed for within-animal statistical analysis and reduced the total number of animals required.

### Drugs

For locomotor activity, 1 mg/kg methamphetamine ((+)-Methamphetamine hydrochloride, National Measurement Institute, NSW, Australia) was administered s.c. 30 min after placing the rat in the chamber. Apomorphine (0.1 mg/kg, R-(−)-apomorphine hydrochloride hemihydrate, Sigma) or methamphetamine (1 mg/kg) were administered s.c. 10 min prior to testing PPI. Drugs were dissolved in saline and administered in a volume of 1 ml/kg. A limitation of this study is that only one dose of each drug was used; however, the selected dose was expected to disrupt PPI and/or induce hyperactivity, based on our previous findings [33], and on preliminary dose-response experiments (see data in Figshare).

### Statistical analysis

All data were expressed as mean ± standard error of the mean (SEM) and analysed using SPSS Statistics 23 (IBM, IL, USA). Body weight, uterus weight, and pituitary gland weight were analysed with one-way analysis of variance (ANOVA) for the 6 groups (SHAM, OVX, 17β, 17α, RAL, TAM), with Bonferroni correction applied for multiple comparisons.

For locomotor activity, i.e. distance travelled, the 5 min interval during which rats were injected was excluded from data analysis. For distance travelled post-injection, a 6 group × 2 drug (saline, methamphetamine) × 3 time (30 min blocks in the 90 min post-injection) repeated-measures ANOVA was used. Main effects of time were always observed and will not be reported unless there were relevant interactions with other factors. Significant group x drug interactions were further explored using pairwise ANOVA comparing the untreated OVX group and the other group of interest, rather than comparing saline and drug within a group because all rat groups showed a methamphetamine-induced hyperactivity. To simplify data presentation, only total distance travelled is presented.

For PPI, a 6 group × 2 drug (saline, methamphetamine; or saline, apomorphine) × 5 prepulse intensities (PP; 2, 4, 8, 12, 16) repeated-measures ANOVA was used. For startle amplitude, a 6 group × 2 drug × 4 block (four blocks of eight 115 dB pulse-alone trials) repeated-measures ANOVA was used. Main effects of PP and block were always observed and will not be reported unless there were relevant interactions with other factors. Significant group x drug interactions were further explored by comparing saline and drug treatments within that group, rather than using pairwise ANOVAs comparing to the untreated OVX group because OVX rats showed a reduction in baseline PPI. ANOVAs including all three drugs were analysed (not reported) and following a significant main effect of drug, further ANOVA was done separated by drug (as described above). To simplify data presentation, the average of the five PP is shown in the figures.

## Results

### Body, uterus, and pituitary gland weight

There were no significant differences in body weight at the time of surgery, however there was a main effect of group by the end of the experiment (weight gain, *F*(5,57) = 5.3, *p* < 0.001; Table 1). Compared to the untreated OVX rats, TAM-treated OVX rats had reduced weight gain (*p* = 0.002). Additionally, there were significant differences in uterus weight between the 6 groups (UW, *F*(5,57) = 61.8, *p* < 0.001; UW/BW, *F*(5,57) = 66.9, *p* < 0.001). Uterus weight itself or as a ratio of body weight was significantly greater in the 17β-treated OVX rats (*p* < 0.001) and the SHAM rats (*p* < 0.001) compared to untreated OVX rats. Uterus weight in RAL, TAM, and 17α-treated OVX rats did not significantly differ from untreated OVX or each other. Pituitary gland weight was different between groups (*F*(5,23) = 3.9, *p* = 0.009). Pituitary weight in the 17β-treated OVX rats was significantly larger compared to the untreated OVX rats (*p* = 0.04) but there were no differences in any other groups.

### Locomotor hyperactivity

**Fig 1:**
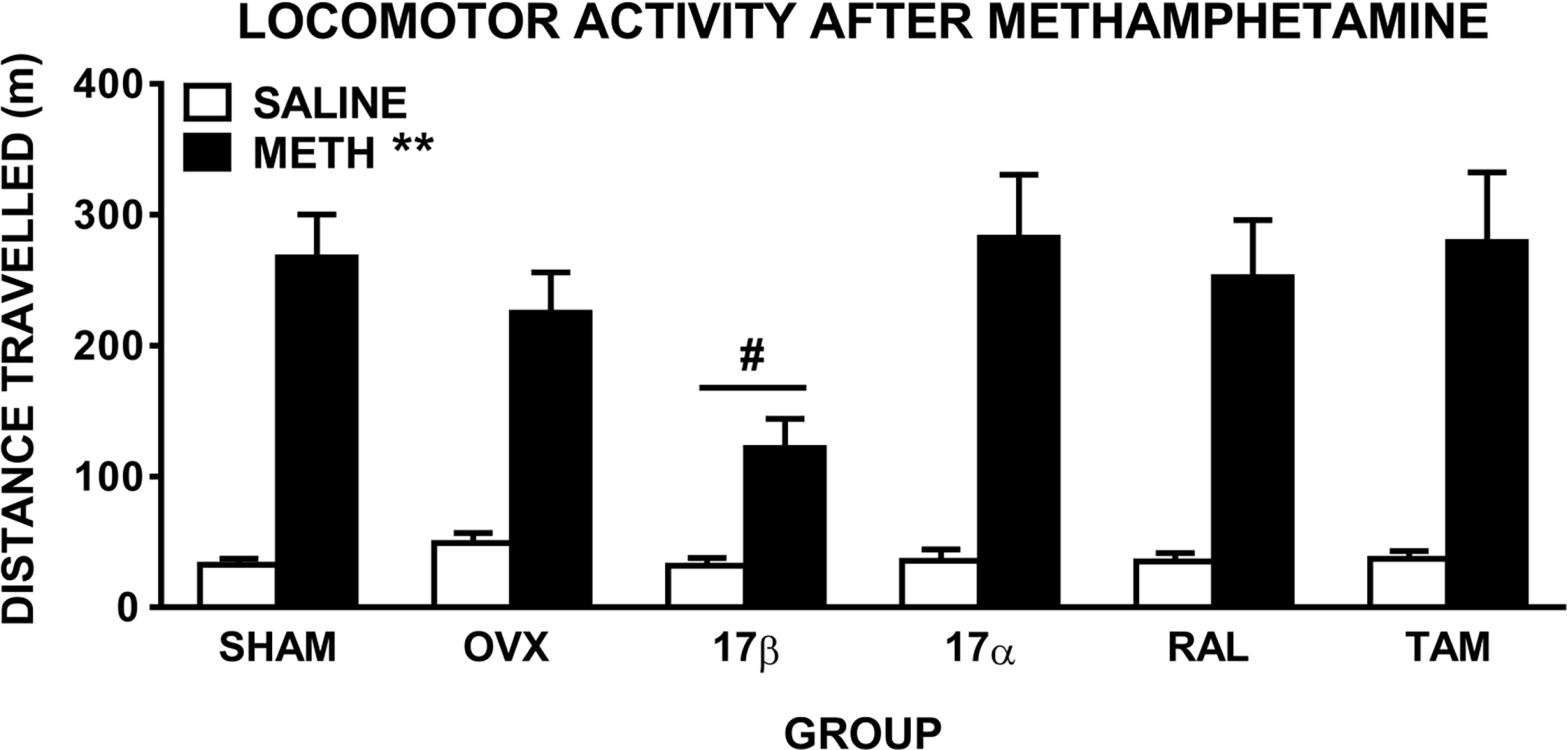
Locomotor activity of female rats displayed as total distance travelled (± SEM) in the 90 min post-administration of methamphetamine (1 mg/kg). Rats were sham-operated (SHAM) rats, untreated ovariectomised (OVX) rats, or OVX rats treated with 17β-estradiol (17β), 17α-estradiol (17α), raloxifene (RAL) or tamoxifen (TAM) (n = 10–11 per group). ** *p* ≤ 0.001 compared to saline (main effect of drug), # *p* = 0.03 compared to OVX group (drug x group interaction).

ANOVA comparing distance travelled during the 90 min post-injection in the 6 groups administered saline and 1 mg/kg methamphetamine revealed there was a significant main effect of drug (*F*(1,57) = 169.6, *p* ≤ 0.001), and a drug x time interaction (*F*(2,114) = 94.9, *p* ≤ 0.001), reflecting the expected increase in distance travelled after methamphetamine, compared to saline, treatment in all groups (Fig 1). When comparing groups after saline injection only, there were no significant main effects or interactions, reflecting no overall group differences in baseline locomotor activity. There was also a significant drug x group interaction (*F*(5,57) = 2.5, *p* = 0.04), suggesting a differential locomotor response between groups after methamphetamine injection. Further ANOVA comparing OVX and 17β showed significantly reduced methamphetamine-induced hyperactivity in 17β-treated OVX rats (drug x group interaction: *F*(1,19) = 5.5, *p* = 0.03; Fig 1). When comparing only methamphetamine treatment in OVX and 17β (2 group x 1 drug x 3 time ANOVA), there was a significant main effect of group (*F*(1, 19) = 7.1, *p* = 0.015), while there was no group difference when comparing saline treatment only (*p* = 0.1). Pairwise comparisons between OVX and each of the other groups showed no significant drug x group interactions, reflecting similar drug-induced hyperactivity between these groups (Fig 1).

### Prepulse inhibition

**Fig 2:**
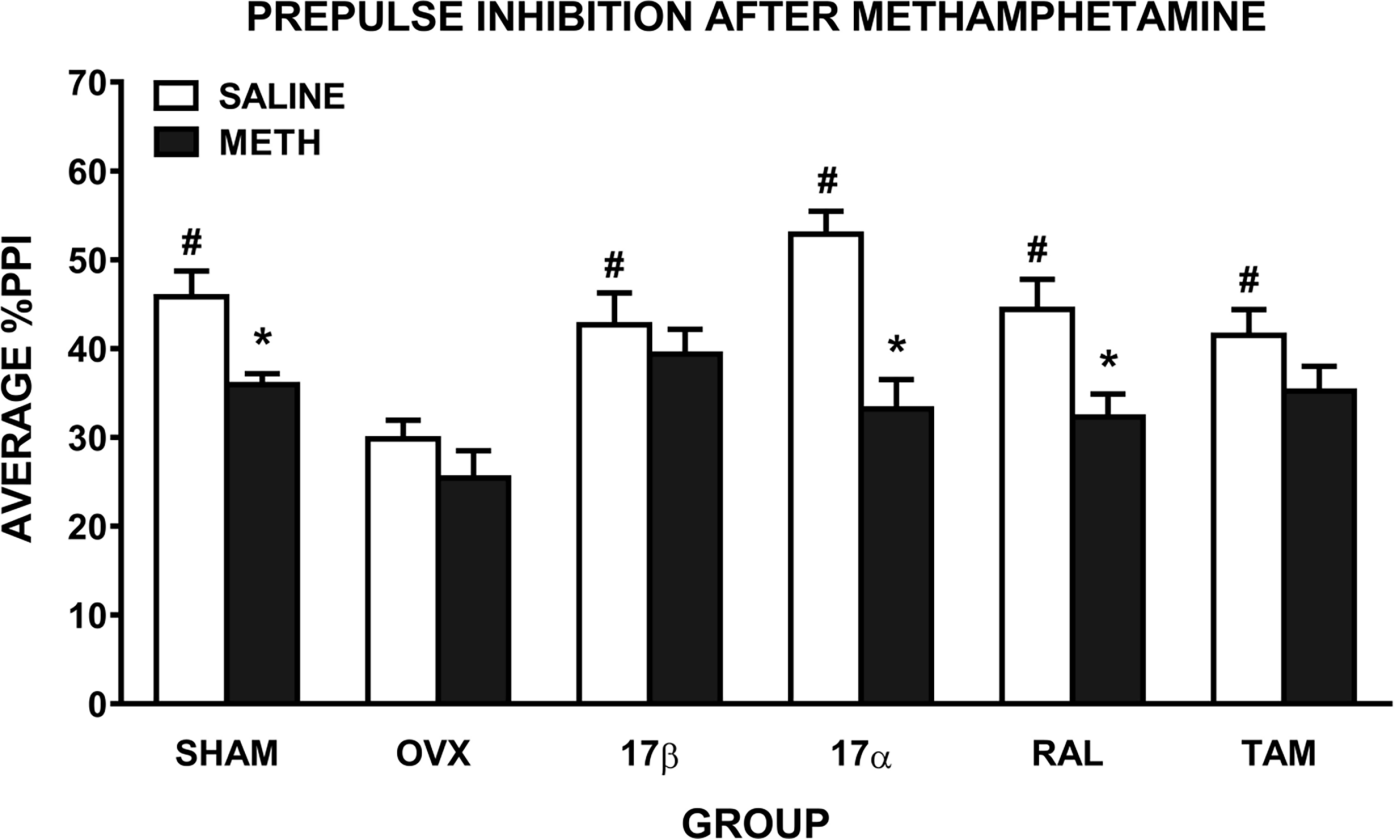
Mean ± SEM %PPI in female rats treated with saline and 1 mg/kg of methamphetamine (METH). Average %PPI reflects the average of the 5 prepulse intensities. Rats were sham-operated (SHAM) rats, untreated ovariectomised (OVX) rats, or OVX rats treated with 17β-estradiol (17β), 17α-estradiol (17α), raloxifene (RAL) or tamoxifen (TAM) (n = 9–11 per group). * *p* ≤ 0.01 compared to saline (main effect of drug); **#** ***p*** ≤ **0.01 compared to OVX group**.

When comparing the effect of saline and methamphetamine on PPI, ANOVA revealed a significant main effect of drug (*F(*1,54) = 46.1, *p* ≤ 0.001), reflecting the expected disruption of PPI after methamphetamine administration, and a drug x group interaction (*F*(5,54) = 3.1, *p* = 0.015). ANOVA comparing the effect of saline treatment on PPI in the 6 groups showed there was a significant main effect of group (*F*(5,54) = 6.2, *p* ≤ 0.001; Fig 2), suggesting a group difference in baseline PPI. Further ANOVAs showed that untreated OVX rats had significantly lower baseline PPI than all other groups (SHAM: *F*(1,19) = 19.1, *p* < 0.001, 17β: *F*(1,18) = 9.5, *p* = 0.007, 17α: *F*(1,17) = 47.1, *p* < 0.001, RAL: *F*(1,17) = 13.5, *p* = 0.002, and TAM: *F*(1,19) = 10.2, *p* = 0.005). ANOVA comparing SHAM rats with all other groups revealed no significant differences in baseline PPI. With respect to the significant drug x group interaction, reflecting differential effects of methamphetamine on PPI between the groups, further ANOVAs were conducted. In untreated OVX rats, compared to saline, there was no significant disruption of PPI after methamphetamine (Fig 2). In contrast, SHAM rats showed a significant disruption of PPI after methamphetamine (*F*(1,10) = 15.9, *p* = 0.003), as did 17α-treated (*F*(1,8) = 41.3, *p* ≤ 0.001) and RAL-treated OVX rats (*F*(1,8) = 22.4, *p* ≤ 0.001). There was no significant effect of methamphetamine on PPI in 17β- or TAM-treated OVX rats (Fig 2). To take into account the OVX-induced reduction in baseline PPI, we also compared only methamphetamine treatment across the groups (6 group x 1 drug x 5 prepulse intensities ANOVA). There was a main effect of group (*F*(5, 54) = 3.1, *p* = 0.016); subsequent pairwise comparisons revealed that untreated OVX rats had reduced PPI after methamphetamine compared to SHAM (*F*(1, 19) = 10.7, *p* = 0.004), 17β (*F*(1, 18) = 11.1, *p* = 0.004) and TAM (*F*(1, 19) = 5.7, *p* = 0.028) rats. This further supports that 17β and TAM treatment can attenuate methamphetamine-induced disruptions of PPI.

**Fig 3:**
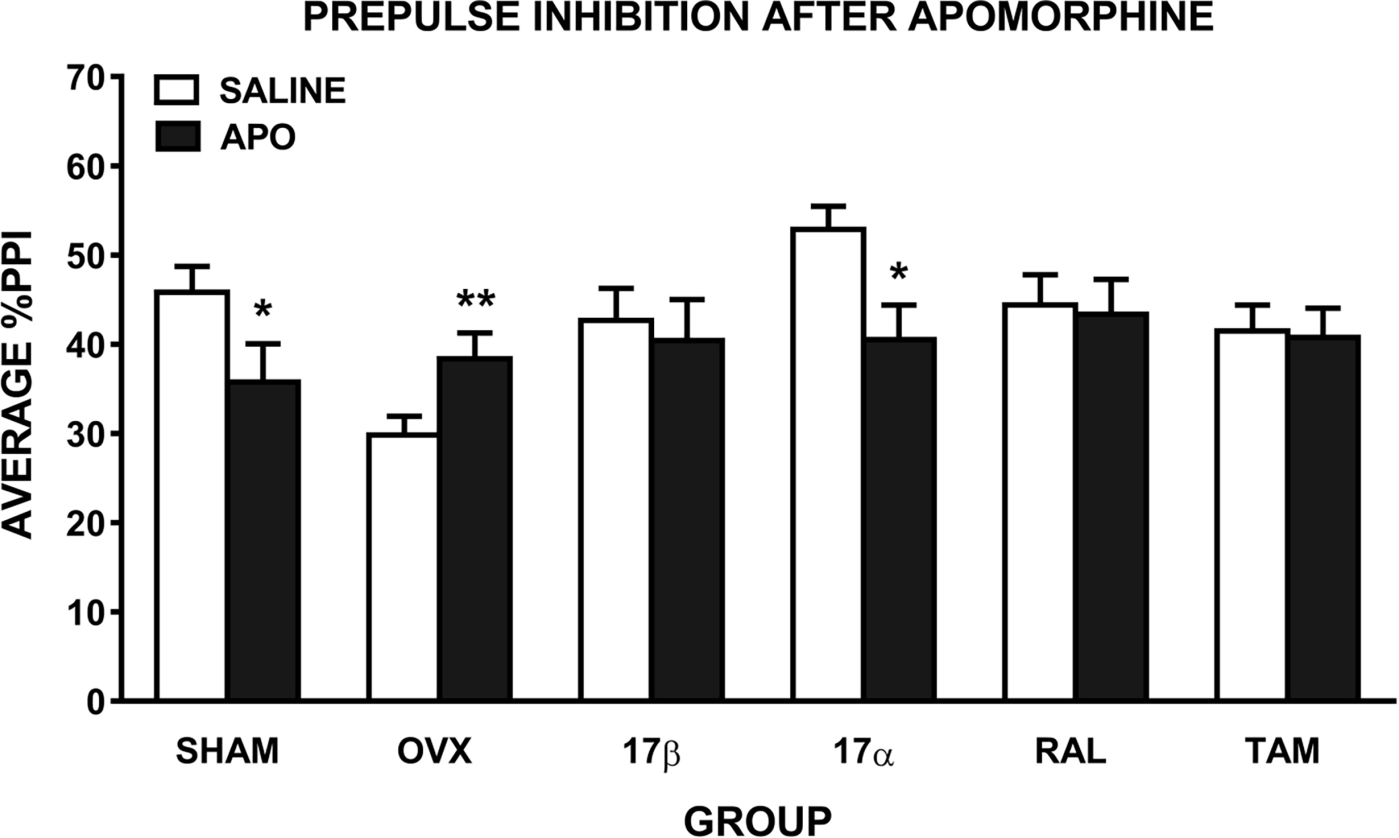
Mean ± SEM %PPI in female rats treated with saline and 0.1 mg/kg of apomorphine (APO). Average %PPI reflects the average of the 5 prepulse intensities. Rats were sham-operated (SHAM) rats, untreated ovariectomised (OVX) rats, or OVX rats treated with 17β-estradiol (17β), 17α-estradiol (17α), raloxifene (RAL) or tamoxifen (TAM) (n = 9–11 per group). ** *p* ≤ 0.001, * *p* ≤ 0.05 compared to saline (main effect of drug).

Analysis of the effect of apomorphine on PPI revealed a trend for a main effect of drug (*F*(1,54) = 3.4, *p* = 0.07), a significant drug x group interaction (*F*(5, 54) = 3.5, *p* = 0.008), and a group x PP interaction (*F*(20,216) = 1.7, *p* = 0.03). Compared to saline, there was a significant disruption of PPI following apomorphine in SHAM (*F*(1,10) = 6.7, *p* = 0.03) and 17α-treated OVX (*F*(1,8) = 5.5, *p* = 0.05) rats, but a significant increase in PPI in untreated OVX rats (*F*(1,9) = 20.7, *p* = 0.001). However, OVX rats treated with 17β, RAL and TAM showed no disruption of PPI following apomorphine administration (Fig 3). To take into account the OVX-induced reduction in baseline PPI, we also compared only apomorphine treatment across the groups (6 group x 1 drug x 5 prepulse intensities ANOVA). Unlike after methamphetamine treatment, PPI after apomorphine treatment was not significantly different across the groups (*p* = 0.8).

When comparing baseline startle responses of all 6 groups after saline treatment, there were no significant main effects or interactions, suggesting a similar startle response in all groups. There were also no significant effects of methamphetamine on startle amplitudes in any of the groups. There was a significant main effect of apomorphine (*F*(1,54) = 9.6, *p* = 0.003), however no interaction with group, reflecting a decrease in startle amplitude after apomorphine administration in all groups (Table 2).

**Table 2:** Mean ± SEM startle amplitude in female rats.

Group	Saline	Methamphetamine	Apomorphine*
SHAM	241.8 ± 14.1	236.7 ± 15.1	199.3 ± 14.8
OVX	287.1 ± 26.5	272.6 ± 29.6	273.2 ± 27.2
17β	231.7 ± 30.0	255.0 ± 51.3	200.7 ± 29.3
17α	287.4 ± 23.7	267.8 ± 12.5	242.4 ± 23.1
RAL	225.4 ± 21.0	253.5 ± 30.2	250.1 ± 32.7
TAM	273.4 ± 22.1	202.4 ± 26.8	232.4 ± 15.3

Rats were treated with saline, 1 mg/kg methamphetamine, and 0.1 mg/kg apomorphine. Rats were sham-operated (SHAM), untreated ovariectomised (OVX), or OVX rats treated with 17β-estradiol (17β), 17α-estradiol (17α), raloxifene (RAL) or tamoxifen (TAM) (n = 9–11 per group).

* *p* ≤ 0.01 compared to saline (main effect of drug).

## Discussion

The aim of this study was to investigate the protective effect of two estradiols, 17β and 17α, and two SERMs, RAL and TAM, against psychotomimetic drug-induced locomotor hyperactivity and disruption of PPI. The key findings were: 1) 17β attenuated locomotor hyperactivity induced by methamphetamine; 2) 17β and TAM attenuated methamphetamine-induced PPI disruption; 3) 17β, RAL and TAM attenuated apomorphine-induced PPI disruption; 4) OVX induced a disruption of baseline PPI that was prevented by the chronic treatment with all estrogenic compounds.

Consistent with our previous studies [13,34], we found a reduction in uterus weight following OVX (-60% compared to SHAM rats); in contrast, we did not find an increase in body weight as typically demonstrated in OVX Sprague-Dawley (SD) rats [13,34]. As expected, 17β significantly reversed the effect of OVX on uterus weight, and increased pituitary gland weight; treatment with 17α, RAL, and TAM did not affect uterus or pituitary weight. These findings are consistent with previous studies showing that 17β treatment, but not the SERMs [34,35], increased uterus [13] and pituitary gland weight [35].

### 17β attenuates methamphetamine-induced locomotor hyperactivity

The current study found that chronic 17β treatment in female OVX LE rats attenuated locomotor hyperactivity induced by methamphetamine, however since ovariectomy did not affect methamphetamine-induced hyperactivity, the reduction in hyperactivity after 17β is not attributed to reversing an OVX-induced effect. 17α, RAL and TAM had no effect on methamphetamine-induced locomotor hyperactivity, highlighting that 17β was the most effective estrogenic compound for attenuating psychotomimetic drug-induced locomotor hyperactivity. To our knowledge, no other study has investigated methamphetamine-induced locomotor hyperactivity in OVX LE rats. Our previous studies in female OVX SD rats found no effect of chronic 17β treatment on amphetamine-induced hyperactivity [33]. In intact male SD rats, TAM treatment attenuated amphetamine-induced hyperactivity [36,37], while RAL treatment significantly increased amphetamine-induced hyperactivity [38]. In intact female SD rats, however, RAL has been shown to attenuate cocaine-induced locomotor hyperactivity [39]. When comparing results across studies using different rat strains it is important to take into account that compared to the SD strain, LE rats express higher levels of catechol-O-methyl transferase (COMT) expression—an enzyme involved in the degradation of catecholamine neurotransmitters including dopamine—in the nucleus accumbens, medial prefrontal cortex, and ventral hippocampus [40]. Moreover, compared to SD rats, LE rats show less sensitivity to disruption of PPI by dopamine receptor agonists, greater dopaminergic-induced Fos expression in the caudate putamen and nucleus accumbens, and differential dopamine-relevant gene expression in the nucleus accumbens [41,42]. Methamphetamine’s action includes releasing catecholamines, such as increasing dopamine release via direct and indirect actions on the dopamine transporter [43–45]; our results suggest that 17β, but not 17α, RAL or TAM, acts to inhibit the action of methamphetamine. It is well established that 17β can modulate the activity of neurotransmitter systems, including altering levels of dopamine receptors (pre- and post-synaptic), transporters, and turnover in cortical and striatal regions [1]. We speculate that the inhibitory action of 17β on methamphetamine-induced hyperactivity is by opposing methamphetamine’s effects on the dopamine transporter [46–48], however the exact mechanism is unclear.

### 17β and TAM attenuate drug-induced disruption of PPI, RAL attenuates apomorphine-induced disruption

Similar to locomotor hyperactivity, 17β treatment attenuated the effect of methamphetamine on PPI, i.e. methamphetamine induced a disruption of PPI in SHAM rats but not 17β-treated rats. Moreover, 17β treatment increased methamphetamine-induced PPI compared to OVX rats. In terms of the effect of apomorphine on PPI, SHAM rats showed the expected disruption of PPI but apomorphine treatment did not disrupt PPI in 17β-treated rats. The results on the effects of the other estrogenic compounds were that, TAM treatment exerted similar effects to 17β in PPI, RAL treatment had more modest effects—only attenuating the apomorphine-induced disruption of PPI, and 17α had no effect on the drug-induced disruptions of PPI. Our findings are consistent with our previous research in SD rats, where apomorphine-induced PPI disruption was attenuated by 17β, TAM and RAL [13]. There are no other studies examining the effects of estrogenic compounds on methamphetamine-induced disruptions of PPI. One study conducted in male mice, found that amphetamine-induced PPI disruption could be reversed by acute treatment with an ER-β agonist [49].

Given that dopamine is the common primary neurotransmitter target of apomorphine and methamphetamine, it is likely that dopaminergic mechanisms are mediating the effects of 17β and TAM. Using the same chronic treatment regimen as in the current study, we previously showed that 17β reversed the OVX-induced increase in dopamine D2 receptors and reduction in dopamine transporter density in the nucleus accumbens [50]. Others found that TAM and RAL had no effect on dopamine D2 receptor binding density in the nucleus accumbens [51], however, TAM and RAL increased dopamine transporter binding in certain subregions of the striatum [48]. Further, they suggest that ER-β mediates these changes in striatal dopamine transporter [48]. In contrast to 17β and TAM, in the current study, RAL did not significantly attenuate methamphetamine-induced PPI disruption. While the exact mechanism of action of SERMs is unclear, it is known that their action can vary depending on the target tissue, ER conformation on ligand binding, and the ratio of ER-α to ER-β [52]. Moreover, TAM has 3-fold greater selectivity for ER-β, while RAL has 20-fold greater selectivity for ER-α [53], and it is possible that ER-β plays a greater role in mediating the ability of estrogenic compounds to attenuate dopamine-induced disruptions of PPI [48,49]. One limitation of this research is the inability to measure the bioavailability in the brain of these estrogenic compounds. Regardless of the exact mechanism, our results confirm that 17β is an effective compound in attenuating dopaminergic drug-induced disruption of PPI, and that the SERM, TAM, was also effective.

### OVX-induced disruption of baseline PPI is reversed by all estrogenic compounds

To our knowledge, this is the first study investigating PPI in OVX LE rats. It was surprising to find that OVX caused a disruption of PPI in LE rats, as we have not observed this effect in our previous studies in OVX SD rats [13,34], nor did OVX have an effect on locomotor activity. We suggest that LE rats may be more sensitive to hormonal modification in PPI than SD rats; for example, some studies found that estrous cycle phase altered PPI in LE rats but not SD rats [54,55]. Given the numerous alterations seen in the brain following OVX [56], it is reasonable to expect changes in behaviour, such as the disruption in baseline PPI that we observed in the current study. For example, OVX results in a substantial loss of dopaminergic cells and reduction of dopamine concentration in the striatum [57,58], and reduced striatal dopamine transporter binding density [46,48]. Importantly, all estrogenic compounds were able to reverse the OVX-induced disruption of baseline PPI, suggesting that removal of the ovaries results in a loss of circulating estrogens that are critical for the regulation of PPI under basal conditions, at least in LE rats. Furthermore, the estrogenic regulation of baseline PPI differs from dopaminergic-mediated PPI, where only some compounds could reverse the dopamine-induced PPI disruptions. The current study found that treatment with 17α rescued baseline PPI in OVX rats, but had no effect on modulating drug-induced PPI disruption or locomotor hyperactivity, suggesting that 17α has a distinct mechanism of action compared to 17β [59]. In contrast to 17β, which has greater affinity for the classical nuclear receptors, ER-α and ER-β, 17α is the preferred ligand of a novel membrane ER, ER-X [59]. It is tempting to speculate that baseline PPI can be rescued by stimulation of ER-X while dopamine-mediated disruption of PPI may require activation of ER-α and ER-β, however, further studies are needed.

In SHAM rats, our data are consistent with previous studies in both the SD and LE strain demonstrating apomorphine-induced disruption of PPI [29,60]. However, in the OVX group only, we observed an apomorphine-induced increase in PPI. One study showed that compared to SD rats, LE rats have decreased sensitivity to dopaminergic disruption of PPI using apomorphine [42]. Together with an OVX-induced decrease in baseline PPI, administration of apomorphine may then increase PPI. We previously showed that the level of baseline PPI can influence the direction of drug effects, such that in rats with low baseline PPI, the serotonin-1A receptor agonist, 8-OH-DPAT, increased PPI, despite this drug typically causing a disruption of PPI [61]. A recent PET study in humans has indeed shown that regulation of dopamine synthesis capacity by apomorphine depends on baseline synthesis capacity, finding an increase in dopamine synthesis in participants with low baseline, and a decrease in those with high baseline [62]. Additional studies are required to improve our understanding of the strain-dependent OVX and apomorphine effects on PPI.

### Conclusion

The current study demonstrated that 17β treatment significantly protected against PPI disruption induced by the indirect dopamine receptor agonist, methamphetamine, and the dopamine D1/D2 receptor agonist, apomorphine, in addition to attenuating methamphetamine-induced locomotor hyperactivity. TAM also attenuated drug-induced disruption of PPI, while RAL only attenuated apomorphine-induced disruption, but neither SERM attenuated drug-induced hyperlocomotion. We found that the brain-synthesized isomer of 17β, 17α, was effective in reversing the OVX-induced disruption of baseline PPI, yet was not protective against dopaminergic-mediated behaviours. This research highlights the utility of some estrogenic compounds to attenuate psychosis-like behaviour in rats. Our findings confirm that 17β is the most effective compound and add to the current literature suggesting that estrogens have therapeutic potential for psychotic disorders.

## References

[1] Gogos A, Sbisa AM, Sun J, Gibbons A, Udawela M, Dean B. A role for estrogen in schizophrenia: clinical and preclinical findings. Int J Endocrinol. 2015;2015 doi: 10.1155/2015/615356 26491441 10.1155/2015/615356 PMC4600562

[2] Kulkarni J, Berk M, Gavrilidis E, Fitzgerald P, Wang W, Worsley R, et al Estradiol for treatment-resistant schizophrenia: A large-scale randomized-controlled trial in women of child-bearing age. Mol Psychiatry. 2015;20(6): 695–702. doi: 10.1038/mp.2014.33 24732671 10.1038/mp.2014.33

[3] Sbisa AM, van den Buuse M, Gogos A. The Effect of 17β-Estradiol and Its Analogues on Cognition in Preclinical and Clinical Research: Relevance to Schizophrenia Psychiatry and Neuroscience Update-Vol II. Cham: Springer International Publishing; 2017 pp. 355–74.

[4] Akhondzadeh S, Nejatisafa AA, Amini H, Mohammadi MR, Larijani B, Kashani L, et al Adjunctive estrogen treatment in women with chronic schizophrenia: a double-blind, randomized, and placebo-controlled trial. Prog Neuropsychopharmacol Biol Psychiatry. 2003;27(6): 1007–12. doi: 10.1016/S0278-5846(03)00161-1 14499318 10.1016/S0278-5846(03)00161-1

[5] Daly E, Vessey MP, Hawkins MM, Carson JL, Gough P, Marsh S. Risk of venous thromboembolism in users of hormone replacement therapy. Lancet Lond Engl. 1996;348(9033): 977–80. doi: 10.1016/S0140-6736(96)07113-9 10.1016/S0140-6736(96)07113-9 8855852

[6] Grodstein F, Stampfer MJ, Goldhaber SZ, Manson JE, Colditz GA, Speizer FE, et al Prospective study of exogenous hormones and risk of pulmonary embolism in women. Lancet Lond Engl. 1996;348(9033): 983–87. doi: 10.1016/S0140-6736(96)07308-4 10.1016/S0140-6736(96)07308-4 8855854

[7] Arevalo MA, Santos-Galindo M, Lagunas N, Azcoitia I, Garcia-Segura LM. Selective estrogen receptor modulators as brain therapeutic agents. J Mol Endocrinol. 2011;46(1): R1–9. doi: 10.1677/JME-10-0122 21071476 10.1677/JME-10-0122

[8] Kulkarni J, Gurvich C, Lee SJ, Gilbert H, Gavrilidis E, de Castella A, et al Piloting the effective therapeutic dose of adjunctive selective estrogen receptor modulator treatment in postmenopausal women with schizophrenia. Psychoneuroendocrinology. 2010;35(8): 1142–47. doi: 10.1016/j.psyneuen.2010.01.014 20171784 10.1016/j.psyneuen.2010.01.014

[9] Usall J, Huerta-Ramos E, Iniesta R, Cobo J, Araya S, Roca M, et al Raloxifene as an adjunctive treatment for postmenopausal women with schizophrenia: A double-blind, randomized, placebo-controlled trial. J Clin Psychiatry. 2011;72(11): 1552 doi: 10.4088/JCP.10m06610 21903021 10.4088/JCP.10m06610

[10] Weickert TW, Weinberg D, Lenroot R, Catts SV, Wells R, Vercammen A, et al Adjunctive raloxifene treatment improves attention and memory in men and women with schizophrenia. Mol Psychiatry. 2015;20(6): 685–94. doi: 10.1038/mp.2015.11 25980345 10.1038/mp.2015.11 PMC4444978

[11] Kianimehr G, Fatehi F, Hashempoor S, Khodaei-Ardakani M-R, Rezaei F, Nazari A, et al Raloxifene adjunctive therapy for postmenopausal women suffering from chronic schizophrenia: A randomized double-blind and placebo controlled trial. DARU J Pharm Sci. 2014;22(1): 55 doi: 10.1186/2008-2231-22-55 25012765 10.1186/2008-2231-22-55 PMC4100751

[12] Huerta-Ramos E, Iniesta R, Ochoa S, Cobo J, Miquel E, Roca M, et al Effects of raloxifene on cognition in postmenopausal women with schizophrenia: A double-blind, randomized, placebo-controlled trial. Eur Neuropsychopharmacol. 2014;24(2): 223–31. doi: 10.1016/j.euroneuro.2013.11.012 24342775 10.1016/j.euroneuro.2013.11.012

[13] Gogos A, van den Buuse M. Comparing the effects of 17β-oestradiol and the selective oestrogen receptor modulators, raloxifene and tamoxifen, on prepulse inhibition in female rats. Schizophr Res. 2015;168(3): 634–39. doi: 10.1016/j.schres.2015.04.029 25979306 10.1016/j.schres.2015.04.029

[14] Kulkarni J, Garland KA, Scaffidi A, Headey B, Anderson R, Castella A de, et al A pilot study of hormone modulation as a new treatment for mania in women with bipolar affective disorder. Psychoneuroendocrinology. 2006;31(4): 543–47. doi: 10.1016/j.psyneuen.2005.11.001 16356651 10.1016/j.psyneuen.2005.11.001

[15] Ikeda T, Makino Y, Yamada MK. 17α-estradiol is generated locally in the male rat brain and can regulate GAD65 expression and anxiety. Neuropharmacology. 2015;90: 9–14. doi: 10.1016/j.neuropharm.2014.10.019 25446575 10.1016/j.neuropharm.2014.10.019

[16] Kaur SP, Bansal S, Chopra K. 17α-estradiol: A candidate neuroserm and non-feminizing estrogen for postmenopausal neuronal complications. Steroids. 2015;96: 7–15. doi: 10.1016/j.steroids.2015.01.004 25595449 10.1016/j.steroids.2015.01.004

[17] Stout MB, Steyn FJ, Jurczak MJ, Camporez J-PG, Zhu Y, Hawse JR, et al 17α-estradiol alleviates age-related metabolic and inflammatory dysfunction in male mice without inducing feminization. J Gerontol A Biol Sci Med Sci. 2016;72(1): 3–15. doi: 10.1093/gerona/glv309 26809497 10.1093/gerona/glv309 PMC5155656

[18] Kuiper G, Carlsson B, Grandien K, Enmark E, Häggblad J, Nilsson S, et al Comparison of the ligand binding specificity and transcript tissue distribution of estrogen receptors α and β. Endocrinology. 1997;138(3): 863–70. doi: 10.1210/endo.138.3.4979 9048584 10.1210/endo.138.3.4979

[19] Toran-Allerand CD, Guan X, MacLusky NJ, Horvath TL, Diano S, Singh M, et al ER-X: A novel, plasma membrane-associated, putative estrogen receptor that is regulated during development and after ischemic brain injury. J Neurosci. 2002;22(19): 8391–401. 12351713 10.1523/JNEUROSCI.22-19-08391.2002 PMC6757764

[20] Gallo D, Zannoni GF, Fabrizi M, Stefano ID, Mantuano E, Scambia G. Comparative effects of 17β-estradiol and phytoestrogens in the regulation of endometrial functions in the rodent uterus. J Endocrinol Invest. 2014;31(1): 48–56. doi: 10.1007/BF03345566 10.1007/BF03345566 18296905

[21] Green PS, Bishop J, Simpkins JW. 17α-estradiol exerts neuroprotective effects on SK-N-SH cells. J Neurosci. 1997;17(2): 511–15. 8987774 10.1523/JNEUROSCI.17-02-00511.1997 PMC6573237

[22] Barha CK, Dalton GL, Galea LAM. Low doses of 17α-estradiol and 17β-estradiol facilitate, whereas higher doses of estrone and 17α- and 17β-estradiol impair, contextual fear conditioning in adult female rats. Neuropsychopharmacology. 2009;35(2): 547–59. 10.1038/npp.2009.161 PMC3055382 19847162

[23] Saravi SSS, Arefidoust A, Yaftian R, Saravi SSS, Dehpour AR. 17α-ethinyl estradiol attenuates depressive-like behavior through GABAA receptor activation/nitrergic pathway blockade in ovariectomized mice. Psychopharmacology (Berl). 2016;233(8): 1467–85. doi: 10.1007/s00213-016-4242-9 26883875 10.1007/s00213-016-4242-9

[24] van den Buuse M. Modeling the positive symptoms of schizophrenia in genetically modified mice: Pharmacology and methodology aspects. Schizophr Bull. 2010;36(2): 246–70. doi: 10.1093/schbul/sbp132 19900963 10.1093/schbul/sbp132 PMC2833124

[25] Gogos A, Kusljic S, Thwaites SJ, van den Buuse M. Sex differences in psychotomimetic-induced behaviours in rats. Behav Brain Res. 2017;322, Part A: 157–66. doi: 10.1016/j.bbr.2017.01.028 28111261 10.1016/j.bbr.2017.01.028

[26] Kumari V, Soni W, Sharma T. Prepulse inhibition of the startle response in risperidone-treated patients: comparison with typical antipsychotics. Schizophr Res. 2002;55(1–2): 139–46. doi: 10.1016/S0920-9964(01)00276-6 11955973 10.1016/s0920-9964(01)00276-6

[27] Ludewig K, Geyer MA, Vollenweider FX. Deficits in prepulse inhibition and habituation in never-medicated, first-episode schizophrenia. Biol Psychiatry. 2003;54(2): 121–28. 12873801 10.1016/s0006-3223(02)01925-x

[28] Geyer MA, Krebs-Thomson K, Braff DL, Swerdlow NR. Pharmacological studies of prepulse inhibition models of sensorimotor gating deficits in schizophrenia: A decade in review. Psychopharmacology (Berl). 2001;156(2–3): 117–54. doi: 10.1007/s002130100811 11549216 10.1007/s002130100811

[29] Gogos A, Kwek P, Chavez C, van den Buuse M. Estrogen treatment blocks 8-Hydroxy-2-Dipropylaminotetralin- and apomorphine-induced disruptions of prepulse inhibition: involvement of dopamine D1 or D2 or serotonin 5-HT1A, 5-HT2A, or 5-HT7 receptors. J Pharmacol Exp Ther. 2010;333(1): 218–27. doi: 10.1124/jpet.109.162123 20042529 10.1124/jpet.109.162123

[30] Gibbs RB. Long-term treatment with estrogen and progesterone enhances acquisition of a spatial memory task by ovariectomized aged rats. Neurobiol Aging. 2000;21(1): 107–16. doi: 10.1016/S0197-4580(00)00103-2 10794855 10.1016/s0197-4580(00)00103-2

[31] Gibbs RB, Johnson DA. Sex-specific effects of gonadectomy and hormone treatment on acquisition of a 12-arm radial maze task by sprague dawley rats. Endocrinology. 2008;149(6): 3176–183. doi: 10.1210/en.2007-1645 18292188 10.1210/en.2007-1645 PMC2408814

[32] Kusljic S, Brosda J, Norman TR, van den Buuse M. Brain serotonin depletion by lesions of the median raphe nucleus enhances the psychotomimetic action of phencyclidine, but not dizocilpine (MK-801), in rats. Brain Res. 2005;1049(2): 217–26. doi: 10.1016/j.brainres.2005.05.017 15953591 10.1016/j.brainres.2005.05.017

[33] Gogos A, Kwek P, van den Buuse M. The role of estrogen and testosterone in female rats in behavioral models of relevance to schizophrenia. Psychopharmacology (Berl). 2012;219(1): 213–24. doi: 10.1007/s00213-011-2389-y 21800043 10.1007/s00213-011-2389-y

[34] Gogos A, van den Buuse M. Estrogen and progesterone prevent disruption of prepulse inhibition by the serotonin-1a receptor agonist 8-hydroxy-2-dipropylaminotetralin. J Pharmacol Exp Ther. 2004;309(1): 267–74. doi: 10.1124/jpet.103.061432 14722325 10.1124/jpet.103.061432

[35] Walf AA, Frye CA. Raloxifene and/or estradiol decrease anxiety-like and depressive-like behavior, whereas only estradiol increases carcinogen-induced tumorigenesis and uterine proliferation among ovariectomized rats. Behav Pharmacol May 2010. 2010;21(3): 231–40. doi: 10.1097/FBP.0b013e32833a5cb0 10.1097/fbp.0b013e32833a5cb0 PMC2885355 20480545

[36] Einat H, Yuan P, Szabo ST, Dogra S, Manji HK. Protein kinase c inhibition by tamoxifen antagonizes manic-like behavior in rats: Implications for the development of novel therapeutics for bipolar disorder. Neuropsychobiology. 2007;55(3–4): 123–31. doi: 10.1159/000106054 17641532 10.1159/000106054

[37] Moretti M, Valvassori SS, Steckert AV, Rochi N, Benedet J, Scaini G, et al Tamoxifen effects on respiratory chain complexes and creatine kinase activities in an animal model of mania. Pharmacol Biochem Behav. 2011;98(2): 304–10. doi: 10.1016/j.pbb.2011.01.017 21281661 10.1016/j.pbb.2011.01.017

[38] Purves-Tyson TD, Boerrigter D, Allen K, Zavitsanou K, Karl T, Djunaidi V, et al Testosterone attenuates and the selective estrogen receptor modulator, raloxifene, potentiates amphetamine-induced locomotion in male rats. Horm Behav. 2015;70: 73–84. doi: 10.1016/j.yhbeh.2015.02.005 25747465 10.1016/j.yhbeh.2015.02.005

[39] Zhang D, Yang S, Yang C, Jin G, Zhen X. Estrogen regulates responses of dopamine neurons in the ventral tegmental area to cocaine. Psychopharmacology (Berl). 2008;199(4): 625–35. doi: 10.1007/s00213-008-1188-6 18516717 10.1007/s00213-008-1188-6

[40] Swerdlow NR, Shilling PD, Breier M, Trim RS, Light GA, Marie RS. Fronto-temporal-mesolimbic gene expression and heritable differences in amphetamine-disrupted sensorimotor gating in rats. Psychopharmacology (Berl). 2012;224(3): 349–62. doi: 10.1007/s00213-012-2758-1 22700037 10.1007/s00213-012-2758-1 PMC5215002

[41] Saint Marie RL, Neary AC, Shoemaker JM, Swerdlow NR. The effects of apomorphine and d-amphetamine on striatal c-Fos expression in Sprague–Dawley and Long Evans rats and their F1 progeny. Brain Res. 2006;1119(1): 203–14. doi: 10.1016/j.brainres.2006.08.045 16979142 10.1016/j.brainres.2006.08.045

[42] Shilling PD, Saint Marie RL, Shoemaker JM, Swerdlow NR. Strain differences in the gating-disruptive effects of apomorphine: Relationship to gene expression in nucleus accumbens signaling pathways. Biol Psychiatry. 2008;63(8): 748–58. doi: 10.1016/j.biopsych.2007.10.015 18083141 10.1016/j.biopsych.2007.10.015 PMC2771724

[43] Fleckenstein AE, Metzger RR, Wilkins DG, Gibb JW, Hanson GR. Rapid and reversible effects of methamphetamine on dopamine transporters. J Pharmacol Exp Ther. 1997;282(2): 834–38. 9262348

[44] Volz TJ, Fleckenstein AE, Hanson GR. Methamphetamine-induced alterations in monoamine transport: implications for neurotoxicity, neuroprotection and treatment. Addiction. 2007;102: 44–48. doi: 10.1111/j.1360-0443.2007.01771.x 17493052 10.1111/j.1360-0443.2007.01771.x

[45] Halpin LE, Collins SA, Yamamoto BK. Neurotoxicity of methamphetamine and 3,4-methylenedioxymethamphetamine. Life Sci. 2014;97(1): 37–44. doi: 10.1016/j.lfs.2013.07.014 23892199 10.1016/j.lfs.2013.07.014 PMC3870191

[46] Bossé R, Rivest R, Di Paolo T. Ovariectomy and estradiol treatment affect the dopamine transporter and its gene expression in the rat brain. Mol Brain Res. 1997;46(1–2): 343–46. doi: 10.1016/S0169-328X(97)00082-X 9191114 10.1016/s0169-328x(97)00082-x

[47] Gardiner SA, Morrison MF, Mozley PD, Mozley LH, Brensinger C, Bilker W, et al Pilot study on the effect of estrogen replacement therapy on brain dopamine transporter availability in healthy, postmenopausal women. Am J Geriatr Psychiatry. 2004;12(6): 621–30. doi: 10.1176/appi.ajgp.12.6.621 15545330 10.1176/appi.ajgp.12.6.621

[48] Le Saux M, Di Paolo T. Influence of oestrogenic compounds on monoamine transporters in rat striatum. J Neuroendocrinol. 2006;18(1): 25–32. doi: 10.1111/j.1365-2826.2005.01380.x 16451217 10.1111/j.1365-2826.2005.01380.x

[49] Labouesse MA, Langhans W, Meyer U. Effects of selective estrogen receptor alpha and beta modulators on prepulse inhibition in male mice. Psychopharmacology (Berl). 2015;232(16): 2981–994. doi: 10.1007/s00213-015-3935-9 25893642 10.1007/s00213-015-3935-9

[50] Chavez C, Hollaus M, Scarr E, Pavey G, Gogos A, van den Buuse M. The effect of estrogen on dopamine and serotonin receptor and transporter levels in the brain: an autoradiography study. Brain Res. 2010;1321: 51–59. doi: 10.1016/j.brainres.2009.12.093 20079719 10.1016/j.brainres.2009.12.093

[51] Landry M, Levesque D, Paolo TD. Estrogenic properties of raloxifene, but not tamoxifen, on D(2) and D(3) dopamine receptors in the rat forebrain. Neuroendocrinology. 2002;76(4): 214–22. doi: 10.1159/000065951 12411738 10.1159/000065951

[52] Labrie F, Labrie C, Bélanger A, Giguere V, Simard J, Mérand Y, et al Pure selective estrogen receptor modulators, new molecules having absolute cell specificity ranging from pure antiestrogenic to complete estrogen-like activities. Adv Protein Chem. 2001;56: 293–368. doi: 10.1016/S0065-3233(01)56009-X 11329857 10.1016/s0065-3233(01)56009-x

[53] Zou A, Marschke KB, Arnold KE, Berger EM, Fitzgerald P, Mais DE, et al Estrogen receptor β activates the human retinoic acid receptorα -1 promoter in response to tamoxifen and other estrogen receptor antagonists, but not in response to estrogen. Mol Endocrinol. 1999;13(3): 418–30. doi: 10.1210/mend.13.3.0253 10076999 10.1210/mend.13.3.0253

[54] Adams AL, Hudson A, Ryan CL, Doucette TA. Effects of estrous stage and time of day on prepulse inhibition in female rats. J Neurosci Methods. 2008;173(2): 295–98. doi: 10.1016/j.jneumeth.2008.06.014 18621080 10.1016/j.jneumeth.2008.06.014

[55] Kinkead B, Yan F, Owens MJ, Nemeroff CB. Endogenous neurotensin is involved in estrous cycle related alterations in prepulse inhibition of the acoustic startle reflex in female rats. Psychoneuroendocrinology. 2008;33(2): 178–87. doi: 10.1016/j.psyneuen.2007.11.005 18155361 10.1016/j.psyneuen.2007.11.005 PMC2254501

[56] Becker JB. Gender differences in dopaminergic function in striatum and nucleus accumbens. Pharmacol Biochem Behav. 1999;64(4): 803–12. doi: 10.1016/S0091-3057(99)00168-9 10593204 10.1016/s0091-3057(99)00168-9

[57] Leranth C, Roth RH, Elsworth JD, Naftolin F, Horvath TL, Redmond DE. Estrogen is essential for maintaining nigrostriatal dopamine neurons in primates: Implications for parkinson’s disease and memory. J Neurosci. 2000;20(23): 8604–609. 11102464 10.1523/JNEUROSCI.20-23-08604.2000 PMC6773080

[58] Xiao L, Becker JB. Quantitative microdialysis determination of extracellular striatal dopamine concentration in male and female rats: Effects of estrous cycle and gonadectomy. Neurosci Lett. 1994;180(2): 155–58. doi: 10.1016/0304-3940(94)90510-X 7700570 10.1016/0304-3940(94)90510-x

[59] Toran-Allerand CD, Tinnikov AA, Singh RJ, Nethrapalli IS. 17alpha-estradiol: A brain-active estrogen? Endocrinology. 2005;146(9): 3843–850. doi: 10.1210/en.2004-1616 15947006 10.1210/en.2004-1616

[60] Swerdlow NR, Breier MR, Marie RLS. Probing the molecular basis for an inherited sensitivity to the startle-gating disruptive effects of apomorphine in rats. Psychopharmacology (Berl). 2011;216(3): 401–10. doi: 10.1007/s00213-011-2228-1 21365203 10.1007/s00213-011-2228-1 PMC5944297

[61] Gogos A, van den Buuse M. The importance of baseline in identifying 8-OH-DPAT-induced effects on prepulse inhibition in rats. Br J Pharmacol. 2007;150(6): 750–57. doi: 10.1038/sj.bjp.0707148 17279088 10.1038/sj.bjp.0707148 PMC2013865

[62] Jauhar S, Veronese M, Rogdaki M, Bloomfield M, Natesan S, Turkheimer F, et al Regulation of dopaminergic function: an [18F]-DOPA PET apomorphine challenge study in humans. Transl Psychiatry. 2017;7(2): e1027 doi: 10.1038/tp.2016.270 28170002 10.1038/tp.2016.270 PMC5438020

